# Poly lactic-co-glycolic acid-based nanoparticles as delivery systems for enhanced cancer immunotherapy

**DOI:** 10.3389/fchem.2022.973666

**Published:** 2022-08-15

**Authors:** Lei Gao, Jing Li, Tianhang Song

**Affiliations:** ^1^ The Second Hospital of Tianjin Medical University, Tianjin, China; ^2^ State Key Laboratory of Elemento-Organic Chemistry, Institute of Elemento-Organic Chemistry, College of Chemistry, Nankai University, Tianjin, China

**Keywords:** PLGA, immunotherapy, adjuvants, antigens, cancer, nanoparticles, drug delivery

## Abstract

Cancer has emerged as one of the most severe diseases in modern times, various therapies have advanced remarkably in recent decades. Unlike the direct therapeutic targeting tumor cells, immunotherapy is a promising strategy that stimulate the immune system. In cancer immunotherapy, polymeric-based nanoparticles can serve as deliver systems for antigens and immunostimulatory molecules, and they have attracted increasing attention and revolutionized cancer therapy. Poly (lactic-co-glycolic acid) (PLGA) is the most frequently used clinically approved biodegradable polymer and has a broad scope of modification of its inherent properties. Recent advances in PLGA based drug delivery systems in cancer immunotherapy have been described in this mini review, with special emphasis on cancer vaccines and tumor microenvironment modulation.

## Introduction

Cancer immunotherapy has received extensive attentions in the past decades, and it has been the fourth most important cancer therapy, after surgery, radiation therapy, and chemotherapy. The rational combination of cancer immunotherapy with other therapeutic modalities has gradually become an emerging therapeutic strategy (Chen et al., 2021). However, mainly due to the lack of effective vectors, many pre-clinical trials have failed to progress to the clinical stage. Nanotechnology offers an opportunity to overcome these limitations. Compared to its bulk structures, nanoparticles have remarkable properties such as smaller size (1–100 nm in diameter), greater surface area to volume ratio, higher cell penetration ability, and enhanced physicochemical properties (Javad et al., 2015). Due to these unique properties, nanoparticles hold great interests in various biomedical applications, and they have also been extensively used as carriers in cancer immunotherapy. As the most widely used immunostimulatory nanoparticles, polymeric nanoparticles are highly appreciated for their preeminent biocompatibility, aqueous solubility, high payload capacity, backbone stability and feasibility of modification to increase targeting ability or responsiveness (Thakur et al., 2020).

The widely developed polymeric nanoparticle-based delivery systems for cancer immunotherapy usually have a core–shell (also known as membrane-core) structure wherein the hydrophobic or charged polymers form the inner core, while the shell-forming polymers have neutral, hydrophilic and flexible properties for stealth nanoparticles. Based on the amphiphilic property, a variety of formulation strategies such as the oil-in-water (O/W) single emulsion process (for hydrophobic cargos) and the water-in-oil-in-water (W/O/W) double emulsion method (for hydrophilic cargos), have been used. Stimuli-responsiveness (temperature, pH, enzymatic, reductive or oxidative, etc.) can be imparted into the core, shell and/or the linkages.

**FIGURE 1:**
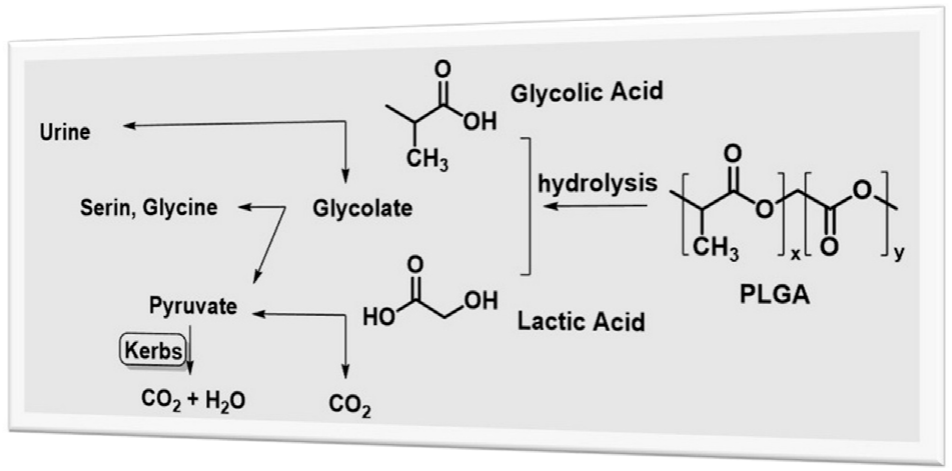
Hydrolysis of PLGA and metabolization of the two monomers.

PLGA (poly-D,L-lactide-co-glycolide) is one of the most successfully used biodegradable polymers for the development of nanomedicines (Acharya and Sahoo, 2011; Rezvantalab et al., 2018; Roointan et al., 2018). Since the body effectively deals with the two biodegradable monomers of PLGA (lactic acid and glycolic acid, which can then be metabolized *via* the Krebs cycle yielding nontoxic byproducts H_2_O and CO_2,_ see Figure 1), it shows very minimal systemic toxicity for drug delivery. A major advantage of PLGA over other polymers is that PLGA has been approved by the U.S. Food and Drug Administration (FDA) and European Medicines Agency (EMA) for pharmaceutical applications *via* parenteral and mucosal routes, leading PLGA-based nanoparticles in a good position for clinical trials (Vasir and Labhasetwar, 2007; Rocha et al., 2022).

**FIGURE 2:**
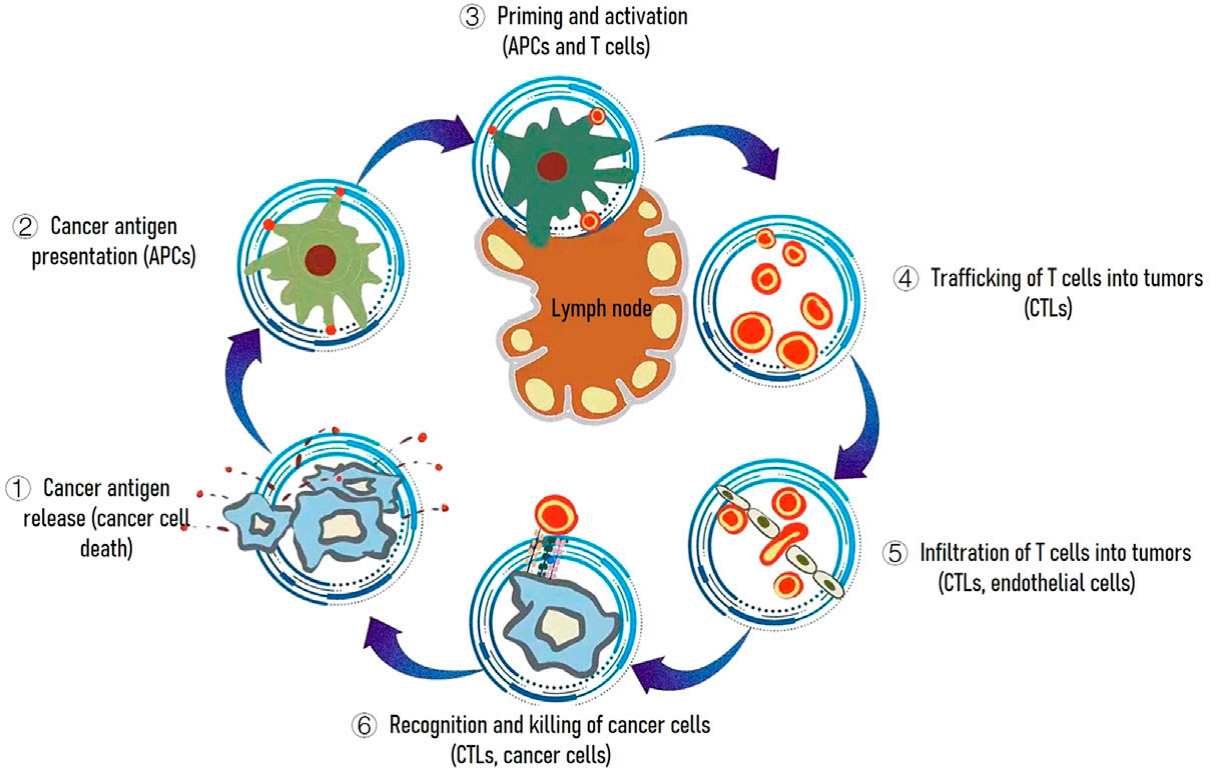
The cancer-immunity cycle. The goal of cancer immunotherapy is to initiate or reinitiate a self-sustaining cycle of cancer immunity. Abbreviations are as follows: APCs, antigen presenting cells; CTLs, cytotoxic T lymphocytes.

Because vaccines can be easy to deploy and have historically represented an approach that has brought enormous medical benefit, therapeutic vaccines against cancer have been explored since the early discovery of tumor-specific antigens (Zhang et al., 2018). In the cancer-immunity cycle, cancer vaccines can primarily promote the cancer antigen presentation step (Chen and Mellman, 2013) (Figure 2) to accelerating and expanding the production of T cell immunity. Although vaccine strategies for the generation of tumor-specific immunological responses continue to have great promise, they were limited on two aspects. First, a general lack of understanding of how to identify proper cancer vaccine delivery system to achieve potent cytotoxic T cell responses. Second, immunosuppressive environment within the tumor resulted in poor therapeutic outcome in clinic. Thus, in this review, we have focused on both cancer vaccines and tumor microenvironment modulation aimed at resulting in an effective systemic antitumor immunity.

## Cancer vaccines

Tumor-associated antigens (TAAs), the adjuvants, and the delivery system are three essential components of therapeutic vaccine (Li et al., 2018). In cancer immunotherapy, PLGA nanoparticles have been tested as delivery systems to ameliorate the efficiencies of therapeutic vaccines. Tumor-associated antigens (TAAs), adjuvants as toll-like-receptor (TLR) agonists, and also tumor lysates have been encapsulated in PLGA nanoparticles (Kohnepoushi et al., 2019). In a study reported by Chen et al. (2016), photothermal agent Indocyanine green (ICG), and Toll-like-receptor-7 agonist imiquimod (R837), were co-encapsulated into PLGA nanoparticles by oil-in-water (o/w) emulsion method. When the multifunctional nanoparticles were used for photothermal ablation of primary tumors, they can generate TAAs, which induce vaccine-like immune responses with R837 as the adjuvant. In combination with the checkpoint-blockade therapy using anti-cytotoxic T-lymphocyte antigen-4 (CTLA4), the generated immunological responses are able to attack the remaining tumor cells in mice, provide inhibition in metastasis and offer a strong immunological memory effect. This strategy showed that combining tumor-specific vaccines that stimulate cytotoxic T lymphocytes (CTL) responses with immune checkpoint blockade therapy is an attractive tool. Recently, Koerner et al. (2021) developed a vaccine based on PLGA particles possess a relatively broad size distribution ranging from nanosized up to 1.5 µm sized particles. Ovalbumin (OVA) and double-stranded (ds) RNA adjuvant Riboxxim were co-encapsulated into the PLGA particles. PLGA particles induced the supreme adjuvant effect of Riboxxim, potently activated murine and human dendritic cells, and elevated tumor-specific CD8^+^ T cell responses. This PLGA particle vaccine delays tumor progression, suppresses tumor metastasis, and provides prolonged survival of immunized mice, and its advantageous therapeutic potency was further enhanced by immune checkpoint blockade that resulted in reinvigoration of cytotoxic T lymphocyte responses and tumor ablation.

Fast recognition and non-specific clearance of nanoparticles by innate immune system, clinical applications of nanoparticles are usually hampered. To further improve the potency, deliver more antigens to the desired site, some modifications of PLGA nanoparticle-based delivery systems were studied. To enhance antigen processing and presentation, the endosomal membrane disrupting agent hydroxychloroquine (HCQ) was encapsulated into PLGA nanoparticles with ovalbumin (OVA) (Liu et al., 2018). *In vitro* experiments showed the nanoparticles enhanced OVA escape from the lysosome into the cytoplasm and also improved cross-presentation of antigen. *In vivo* studies concluded that this co-delivery nanovaccine can provide strong CD8^+^ T cell immune responses that induced tumor cell apoptosis and long-lasting antigen-specific memory immune responses in vaccinated mice. Zhang et al. (2020) have developed a PLGA nanoparticle vaccine in which PLGA nanoparticle as delivery system encapsulated the antigenic peptide HPV16 E7_44-62_ and adenosine triphosphate (ATP) as adjuvant component. Peptides were encapsulated into PLGA nanoparticles using a two-stage emulsification method, ATP was introduced by simple mixing 10 μl ATP with 90 μl of E7_44-62_-loaded nanoparticles. Employing PLGA nanoparticles increased lymph node accumulation, and dendritic cell (DC) uptake of the E7 peptide. ATP adjuvant further increased the migration, nanoparticle uptake, and maturation of DCs. ATP-adjuvanted nanoparticles stimulated cell-mediated immune responses, completely abolished the growth of TC-1 tumors, produced long-lasting immunity and significantly delayed tumor progression in vaccinated mice.

Because of the “proton sponge” effect-induced antigen escape, cationic polymers can facilitate antigen adsorption and uptake by APCs such as DCs (Chen et al., 2014). To endow the PLGA nanoparticles with positive surface charge, a relatively safe cationic surfactant dimethyl-dioctadecyl-ammonium bromide (DDAB) is chosen, and the DDAB/PLGA nanoparticles were prepared using nanoprecipitation (Han et al., 2021). The positively charged surface of the DDAB/PLGA nanoparticles enabled the negatively charged antigen of the model antigen ovalbumin (OVA_257-264_) to be easily absorbed to the surface *via* electrostatic interaction to obtain an OVA@DDAB/PLGA nanovaccine. Experiments performed *in vitro* revealed that the nanovaccine induced antigen escape from lysosome into cytoplasm with 10 times increased cross-presentation activity than naked OVA. The nanovaccine showed excellent draining lymph nodes (LNs) transportation ability by passive lymphatic drainage and active DC transport. After immunization, the OVA@DDAB/PLGA nanovaccine can stimulate both humoral and cell-mediated immune responses and offer a strong immunological memory effect.

Another strategy that exhibits great potential in the disease diagnosis and therapeutics is membrane coating nanotechnology (Liu et al., 2022). It has revolutionized the design of cancer vaccine by endowing targeting, antigen presentation and immune stimulation. Diverse membrane coating platforms have been developed for cancer vaccine design. In a study by Yang et al. (2018), TLR7 agonist imiquimod (R837) loaded PLGA nanoparticles were coated with B16-OVA cancer cell membranes, whose surface proteins could act as tumor specific antigens. The obtained nanoparticles were further modified with mannose by a lipid-anchoring method. This PLGA complex was efficiently targeted and internalized by antigen presenting cells (APCs) such as DCs, which triggered potent antitumor immunotherapeutic efficacy. Yang et al. (2019) designed a lipid-coated PLGA hybrid particles for the co-delivery of mRNA and TLR7 adjuvant. In this carrier system, the PLGA core enabled the efficient loading of the hydrophobic gardiquimod, and the lipid shell loaded the mRNA *via* electrostatic interaction. The hybrid nanovaccine led to the effective antigen expression and DC maturation *in vitro*, also a stronger antigen-specific immune response was obtained. The spatial/temporal overlap of the antigen and adjuvant *via* the core-shell nanoparticles are found to be beneficial for tumor growth inhibition. Zhou et al. (2020) designed a nanovaccine composing of a PLGA core to encapsulate TLR7 agonist imiquimod (R837), a phospholipid membrane to load antigen peptide (αOVA) and apolipoprotein E3 (ApoE3). Incorporation of ApoE3 facilitate nanovaccines uptake through the micropinocytosis pathway, and significantly promoted DCs maturation and antigen presentation. More *in vivo* studies showed that these nanoparticles migrated to the lymph nodes, leading to strong T cell immune responses. The nanovaccine also provided inhibition in metastasis in lung, and exerted superior therapeutic efficiency on B16-OVA tumor-bearing mice when in combination with αPD-1 therapy. Nowadays the membrane-coated technology with membranes from different types of cells offers promising opportunities for cancer immunotherapy, cell membranes employed have gradually shifted from natural to engineered (Chen et al., 2020; Liu et al., 2020). In the nanovaccine developed by Gou et al. (2021), peptide CBP-12 expressed biomimetic cancer cell membrane coating strategy was adopted to specifically target Clec9a^+^ DCs. The membrane coated PLGA drug-delivery system efficiently delivers tumor antigen and STING agonist (cGAMP) to Clec9a^+^ DCs, significantly enhanced IFN-stimulated expression of genes and antigen cross-presentation of Clec9a^+^ DCs, eliciting strong antitumor effects in anti-PD-1-resistant tumor models without obvious cytotoxicity. Moreover, combination of the nanovaccine with radiotherapy remarkably enhances the cancer immunotherapy effects.

## Tumor microenvironment modulation

The immunosuppressive tumor microenvironment consists of cells, soluble factors, signaling molecules, extracellular matrix, and mechanical cues (Swartz et al., 2012), it is created by the tumor and dominated by tumor-induced interactions (Whiteside, 2008). Modulating the tumor microenvironment with efficient modulator will significantly promote the immune responses inside a tumor. Using nanoparticles for remodeling the tumor microenvironment is a promising immunotherapeutic strategy to overcome “immune escape” and resulting in an effective systemic antitumor immunity.

Efficient capture and presentation of tumor antigens by APCs, especially dendritic cells (DCs), are crucial for anti-tumor immunity. However, APCs are immunosuppressed in the tumor microenvironment. Kim et al. (Kim et al., 2018a) showed that incorporating TLR 7/8 agonists into PLGA nanoparticles could significantly increase co-stimulatory molecule expression and antigen presentation in DCs compared to free agonists. *In vivo* studies showed that these nanoparticles migrated to the lymph nodes, triggering DC activation and expansion, leading to enhanced cytotoxic T lymphocytes (CTL) responses, and in turn, improving prophylactic and therapeutic efficacy in melanoma, bladder, and renal cell carcinoma tumor models. Later, to overcome fast clearance from the injection site, pH responsiveness is incorporated into the TLR7/8 agonist delivery platform (Kim et al., 2018b). Bicarbonate salt was adopted, the salt generates carbon dioxide at acidic pH, which can disrupt the polymer shell to rapidly release the payload. The acid-responsive formulation was characterized by higher drug encapsulation and DC activation leading to the expansion of activated natural killer (NK) cells and antigen-specific CD8^+^ T cells. Da Silva et al. (Da Silva et al., 2019) used PLGA nanoparticles as delivery vehicles for the co-delivery of three immune adjuvants [the TLR3 agonist Poly (I:C;pIC), TLR7/8 agonist Resiquimod (R848) and the chemokine Macrophage Inflammatory Protein-3 alpha (MIP3α)] to significantly improve the therapeutic efficacy of cancer vaccines. Co-delivery of these modulating agents using PLGA nanoparticles significantly potentiated the cancer vaccine antitumor effects. The long-term survival of mice with established large carcinoma tumors was improved to 75%–100%, and the progression free survival of the mice nearly doubled. The potent adjuvant effect was associated with lymphoid and myeloid cell population alterations in the tumor and tumor-draining lymph node. Lu et al. (2021) developed a co-delivery immunotherapeutic strategy of the phagocytosis checkpoint (signal regulatory protein α, SIRPα) silencer and stimulator of interferon genes (STING) of APCs. A small interfering RNA targeting SIRPα (siSIRPα) and a STING agonist (cGAMP) were encapsulation into PLGA-based polymeric nanoparticles using the double emulsification method. In the ovalbumin-expressing B16-F10 (OVA-B16-F10) melanoma model, NPsiSIRPa/cGAMP stimulated the activation of OVA-specific CD8^+^ T cells and induced holistic anti-tumor immune responses by reversing the immunosuppressive phenotype of APCs.

As effectors of innate immunity, natural killer (NK) cells represent ‘the first line’ of defense against pathogens and mediate potent antitumor cytotoxicity *in vitro* through secretion of cytotoxic lymphokines and disruption of the tumor vascular. Adoptive immunotherapy (AIT) with natural killer (NK) cells has emerged as a potential treatment strategy. However, their paucity in tumor infiltrates causes the low therapeutic efficacy of NK cell ATI. Park et al. (2017). demonstrate MRI-monitored transcatheter intra-arterial (IA) local delivery of IFN-γ and iron oxide nanocubes (IONC) co-encapsulated PLGA nanoparticles to induce efficient NK cells infiltration to tumor sites for the targeted treatment of liver cancer. In an orthotopic liver tumor VX2 rabbit model, the prepared nanoparticles showed a sustained IFN-γ release and highly sensitive MR T2 contrast effects, significantly increased NK-cell infiltration into the liver tumor site.

Neutrophils can exert antitumoral functions especially in early stage of tumor development. However, they have been also shown to facilitate tumorigenesis and mediate immunosuppression. Due to their innate phagocytic functions and oriented migration capabilities in response to chemoattractants, nanoparticle-loaded neutrophils were used as “Trojan horses” (Hao et al., 2020). The pre-implantation of chemokine *CXCL1*-laden hydrogels could trigger and induce a targeted signal to attract an influx of neutrophils carrying the therapeutic goods to the desired position, thus the effectiveness of neutrophil-mediated nanoparticles drug delivery system is improved. *In vivo* studies showed that the combinatorial regimen of using the paclitaxel (PTX) loaded PLGA nanoparticles with the *CXCL1* chemokine laden PLGA-PEG-PLGA thermosensitive hydrogels exhibited superior tumor inhibition capability in mouse models of melanoma.

Tumor-associated macrophages (TAMs) are abundant in most human and experimental murine cancers, they are the major contributors to tumor angiogenesis, and also influence lymphocyte infiltration, leading to immunosuppression (Balkwill et al., 2012). Zhang et al. (2021) developed an M2-like macrophage-targeting nanoparticles to switch the tumor-promoting immune suppressive microenvironment by reprogramming TAMs. In these nanoparticles resiquimod (R848, a potent driver of macrophage reprogramming) loaded PLGA nanoparticles were coated with the B16-OVA cancer cell membrane. The membrane can increase the expression of CD47, which could avoid the nanoparticles being cleared by the reticuloendothelial system. The membrane was further modified with poly (ethylene glycol) (PEG) to achieve better long blood circulation and finally modified with M2pep to improve the selectivity and specificity for M2-like macrophages. The nanoparticles provide an effective and selective reprogramming strategy for macrophage-mediated cancer immunotherapy. More *in vivo* studies showed that the loading nanoparticles reduced tumor size, and prolonged survival compared to the control groups.

## Conclusion

This article briefly reviewed recent studies directed to improve the efficiency of PLGA-based delivery systems in cancer immunotherapy. In Table 1, we summarized these studies. Biocompatibility, biodegradability, and feasibility of modification are the most interesting features of PLGA-based nanoparticles, which can support these materials to have a good position in the development of nanomedicines. However, the high cost of production, the difficulty of the scale-up, fast *in vivo* degradation of non-coated nanoparticles and the relatively low drug loading efficiency are the main limitations of PLGA-based delivery systems. Although further research and clinical studies are still needed to improve the efficacy of drug delivery, the recent studies presented in this mini review clearly illustrate the promise of PLGA-based nanoparticles for novel treatments of cancer in the future.

**TABLE 1:** PLGA-based nanoparticles used as delivery systems in cancer immunotherapy.

Nanocarrier	Payload	Tumor model	Outcomes	References
PLGA NPs	ICG, R837	4T1 breast cancer, CT26 cancer	Promoted generation of TAAs	Chen et al. (2016)
PLGA NPs	OVA, Riboxxim	EG7-OVA thymoma	Induced strong anti-tumor immune response	Koerner et al. (2021)
PLGA NPs	OVA, Hydroxychloroquine (HCQ)	EG7-OVA thymoma	Provided strong CD8^+^ T cell immune responses	Liu et al. (2018)
PLGA NPs	HPV16 E7_44-62_, ATP	TC-1 tumor	Induced strong anti-tumor immune response	Zhang et al. (2020)
DDAB/PLGA NPs	OVA	--	Enhanced the efficiency of nanovaccine	Han et al. (2021)
Cancer cell membrane-coated PLGA NPs	R837	4T1 breast cancer	Enhanced uptake of vaccine by DCs, which significantly promoted DCs maturation and antigen presentation	Yang et al. (2018)
Engineered peptide-expressed biomimetic cancer cell membrane-coated PLGA NPs	2′3′-cGAMP	B16-OVA melanoma, 4T1 breast cancer		Gou et al. (2021)
Lipid-coated PLGA NPs	mRNA, gardiquimod	B16-OVA melanoma		Yang et al. (2019)
Lipid-coated PLGA NPs	R837, OVA, ApoE3	B16-OVA melanoma		Zhou et al. (2020)
PLGA NPs	TLR7/8 agonists	Melanoma, Bladder, Renal Cell Carcinoma	Enhanced antigen specific immune response	Kim et al. (2018a)
PLGA NPs	TLR7/8 agonists, NaHCO_3_	Melanoma	Resulted in higher loading of payload	Kim et al. (2018b)
PLGA NPs	Poly (I:C), R848, MIP3α	Carcinoma, Lymphoma	Enhanced the efficiency of nanovaccine	Da Silva et al. (2019)
PLGA NPs	siSIRPα, cGAMP	Melanoma	Induced strong anti-tumor immune response	Lu et al. (2021)
PLGA NPs	IFN-γ, Iron oxide nanocubes	Liver tumor	Enabled MRI-guided transcatheter IA delivery to liver tumor	Park et al. (2017)
PLGA NPs	Paclitaxel (PTX)	Melanoma	Enhanced the tumor inhibition capability	Hao et al. (2020)
M2pep modified cancer cell membrane-coated PLGA NPs	R848	B16-OVA melanoma	Inhibited tumor growth by reporamming TAMs	Zhang et al. (2021)
